# Lipopolysaccharide immune stimulation but not β-mannanase supplementation affects maintenance energy requirements in young weaned pigs

**DOI:** 10.1186/s40104-018-0264-y

**Published:** 2018-06-15

**Authors:** Nichole F. Huntley, C. Martin Nyachoti, John F. Patience

**Affiliations:** 1 Department of Animal Science, Iowa State University, Ames, IA 50011 USA; 2 Department of Animal Science, University of Manitoba, 226 Animal Science Building, Winnipeg, MB R3T 2N2 Canada

**Keywords:** Acute phase proteins, β-Mannan, Cytokines, Digestibility, Feed induced immune response, Heat production, Inflammation, Lipopolysaccharide, Nitrogen balance, Swine

## Abstract

**Background:**

Pathogen or diet-induced immune activation can partition energy and nutrients away from growth, but clear relationships between immune responses and the direction and magnitude of energy partitioning responses have yet to be elucidated. The objectives were to determine how β-mannanase supplementation and lipopolysaccharide (LPS) immune stimulation affect maintenance energy requirements (MEm) and to characterize immune parameters, digestibility, growth performance, and energy balance.

**Methods:**

In a randomized complete block design, 30 young weaned pigs were assigned to either the control treatment (CON; basal corn, soybean meal and soybean hulls diet), the enzyme treatment (ENZ; basal diet + 0.056% β-mannanase), or the immune system stimulation treatment (ISS; basal diet + 0.056% β-mannanase, challenged with repeated increasing doses of *Escherichia coli* LPS). The experiment consisted of a 10-d adaptation period, 5-d digestibility and nitrogen balance measurement, 22 h of heat production (HP) measurements, and 12 h of fasting HP measurements in indirect calorimetry chambers. The immune challenge consisted of 4 injections of either LPS (ISS) or sterile saline (CON and ENZ), one every 48 h beginning on d 10. Blood was collected pre- and post-challenge for complete blood counts with differential, haptoglobin and mannan binding lectin, 12 cytokines, and glucose and insulin concentrations.

**Results:**

Beta-mannanase supplementation did not affect immune status, nutrient digestibility, growth performance, energy balance, or ME_m_. The ISS treatment induced fever, elevated proinflammatory cytokines and decreased leukocyte concentrations (*P* < 0.05). The ISS treatment did not impact nitrogen balance or nutrient digestibility (*P* > 0.10), but increased total HP (21%) and ME_m_ (23%), resulting in decreased lipid deposition (−30%) and average daily gain (−18%) (*P* < 0.05).

**Conclusions:**

This experiment provides novel data on β-mannanase supplementation effects on immune parameters and energy balance in pigs and is the first to directly relate decreased ADG to increased ME_m_ independent of changes in feed intake in immune challenged pigs. Immune stimulation increased energy partitioning to the immune system by 23% which limited lipid deposition and weight gain. Understanding energy and nutrient partitioning in immune-stressed pigs may provide insight into more effective feeding and management strategies.

**Electronic supplementary material:**

The online version of this article (10.1186/s40104-018-0264-y) contains supplementary material, which is available to authorized users.

## Background

The negative influence of an immune challenge on animal growth is well established. Pro-inflammatory cytokines orchestrate an immune response resulting in fever, acute phase protein (APP) production, and leukocyte proliferation, each of which requires additional energy and amino acids (AA). Therefore, a perceived immune challenge can theoretically partition energy and nutrients away from productive processes such as muscle growth and negatively impact the efficiency and cost of meat production [1]. Innate immune activation occurs when pathogen-associated molecular patterns are detected such as the lipid-A component of lipopolysaccharide (LPS) from gram-negative bacteria [2]. However, certain dietary components, such as β-mannan in soybean, copra, and palm kernel meals, mimic carbohydrate structures on pathogen surfaces [3] and have previously been shown to activate the innate immune system [4, 5], termed a feed-induced immune response (FIIR).

To inhibit a β-mannan derived FIIR, interest in β-mannanase enzyme supplementation has increased. It is hypothesized that the hydrolyzed manno-oligosaccharides can no longer crosslink and stimulate multiple mannose receptors, thus reducing immune stimulation and associated energy costs. Research in poultry demonstrated that β-mannanase decreased plasma APP concentration and improved growth performance and feed efficiency leading to the conclusion that β-mannanase supplementation spared energy through prevention of the FIIR [6, 7]. In pigs, performance responses to β-mannanase are less consistent than in poultry and reports on immune responses are limited and effects on energy partitioning have yet to be evaluated.

Nutrient partitioning in pigs during a pathogen challenge has received more attention, often utilizing a LPS challenge model [8]. Physiological responses to a LPS challenge in pigs have been well characterized. Similar to disease challenges, LPS induces anorexia, fever, and nutrient repartitioning leading to decreased growth and efficiency [1, 2, 9]. Fever is an energetically expensive process and its effects on sheep and human maintenance energy requirements have been estimated [10]. Immune system activation also significantly shifts glucose metabolism and glucose requirements during an LPS challenge in pigs have been estimated to be approximately 1.1 g/(kg BW^0.75^·h) [11]. Yet few studies have addressed comprehensive changes in energy partitioning during an immune response and clear relationships between measured immune responses and the direction and magnitude of changes in energy partitioning have yet to be elucidated.

Therefore, the objectives of this experiment were to determine how β-mannanase supplementation and innate immune stimulation each affect maintenance energy requirements and to characterize changes in immune parameters, nutrient digestibility, growth performance, and energy balance. We hypothesized that innate immune stimulation would increase maintenance energy requirements by initiating a cytokine-driven febrile response and inflammatory state, and that β-mannanase supplementation would decrease maintenance energy requirements through an energy sparing effect of FIIR prevention.

## Methods

All experimental procedures adhered to guidelines for the ethical and humane use of animals for research and were reviewed and approved by the University of Manitoba Animal Care Committee.

### Animals and experimental design

Thirty growing barrows [(Yorkshire × Landrace) × Duroc] were acquired from the Glenlea Swine Research Unit, University of Manitoba at an average body weight (BW) of 9.60 ± 2.00 kg. The experiment was conducted using a randomized complete block design. Pigs were blocked by weight and randomly assigned to one of three treatments (Table 1). A staggered time course was utilized to accommodate the limited number of calorimetry chambers available, whereby 10 blocks of three pigs each (one pig per treatment) began the experiment 4 d after the previous block. Day one BW was similar among treatments (10.27 ± 0.08 kg).

**Table 1:** Summary of experimental treatments

	Experimental treatment		
	CON^a^	ENZ^b^	ISS^c^
Diet	Control	Control + β-Mannanase	Control + β-Mannanase
β-mannanase inclusion	No	Yes	Yes
Challenge treatment	Saline	Saline	*E. coli* LPS

^a^Control treatment (CON) = pigs fed basal diet with no LPS (*Escherichia coli* serotype O55:B5) injection

^b^Enzyme treatment (ENZ) = pigs fed enzyme diet (0.056% β-mannanase) with no LPS injection

^c^Immune system stimulation treatment (ISS) = pigs fed enzyme diet (0.056% β-mannanase) with LPS injection

### Experimental diets, treatments and procedures

All diets were formulated on the ratio of standardized ileal digestible lysine to metabolizable energy (ME) and met or exceeded all specified nutrient requirements of growing pigs from 11 to 25 kg [12]. Pigs were fed at 2.5 times their maintenance ME requirements [12], once daily at 08:00 h and had free access to water at all times. Pigs were fed a common pre-trial diet (Additional file 1: Table S1) that was corn-soybean meal-based. The experimental basal diet (Table 2) was formulated with high soybean meal and soybean hull inclusion levels to increase dietary β-mannan concentration.

**Table 2:** Experimental diet ingredient and analyzed nutrient composition (as-fed basis)

Item	Control diet	Enzyme diet
Ingredient, % of diet		
Corn	47.33	47.27
Soybean meal, (dehulled, solvent extracted)	38.40	38.40
Soybean hulls	10.00	10.00
Soybean oil	1.85	1.85
Limestone	1.04	1.04
Monocalcium phosphate	0.60	0.60
Vitamin premix^a^	0.33	0.33
Trace mineral premix^b^	0.20	0.20
Salt	0.25	0.25
Hemicell HT-D^c^	0.00	0.06
Calculated composition, % of diet		
SID Lys	1.18	1.18
SID Met	0.32	0.32
SID Thr	0.75	0.75
SID Trp	0.26	0.26
SID Cys + Met	0.62	0.62
β-mannan^e^	1.33	1.33
Analyzed composition, % of diet		
DM	86.75	87.17
GE, Mcal/kg	4.03	4.00
CP	22.28	21.83
EE^d^	4.02	3.97
Starch	29.34	30.89
NDF	12.17	11.83
ADF	6.97	6.79
endo-1,4-β-mannanase^f^, IU/kg	*Below detectable limit* ^g^	150,000

^a^Provided per kilogram of complete diet: 6,614 IU of vitamin A; 827 IU of vitamin D; 26 IU of vitamin E; 2.6 mg of vitamin K; 29.8 mg of niacin; 16.5 mg of pantothenic acid; 5.0 mg of riboflavin; 0.023 mg of vitamin B_12_

^b^Provided per kilogram of complete diet: Zn, 165 mg as ZnSO_4_; Fe, 165 mg as FeSO_4_; Mn, 39 mg as MnSO_4_; Cu, 17 mg as CuSO_4_; I, 0.3 mg as Ca (IO_3_)_2_; and Se, 0.3 mg as Na_2_SeO_3_

^c^Hemicell™ HT-D, Elanco Animal Health, Guelph, ON, Canada; endo-1,4-β-mannanase (160 × 10^6^ units/kg) from *Paenibacillus alvei*

^d^Acid hydrolyzed ether extract

^e^β-mannan concentration was calculated using values reported in Shastak et al. [70]

^f^endo-1,4-β-mannanase activity. 1 IU = the amount of enzyme which generates 0.72 micrograms of reducing sugars per minute from a mannose-containing substrate at pH 7.0 and temperature of 40 °C

^g^The lowest detectable limit was 15,000 IU/kg

Due to the availability of three indirect calorimetry chambers, three experimental treatments were evaluated (Table 1). The control treatment (CON) received the basal diet, while the enzyme treatment (ENZ) received CON supplemented with 0.056% β-mannanase (Hemicell™ HT-D, Elanco Animal Health, Guelph, ON, Canada; endo-1,4-β-mannanase (160 × 10^6^ units/kg) from *Paenibacillus alvei*). The third treatment was challenged with repeated LPS immune system stimulation (ISS) and received the same diet as ENZ. This treatment design was determined based on the hypothesis, supported by previous research, that β-mannanase would inhibit a FIIR if it occurred in CON [6, 7]. In this way, the effect of an innate immune stimulation by LPS could be evaluated independent of a FIIR.

Upon arrival and during the pre-trial period, pigs were housed individually in pens (1.83 m × 1.22 m) with plastic-covered expanded metal flooring in a temperature-controlled room (26 ± 2 °C). Daily feed allotment during the pre-trial period was adjusted based on BW measured every 4 d. Pigs were maintained on the pre-trial diet for at least 4 d until initiation of the experiment for their respective block, at which time pigs received their assigned treatment diets. The experiment consisted of a 10-d adaptation phase, a 5-d total feces and urine collection phase, and 34 h of heat production (HP) measurements.

At trial initiation (d 1), pigs were individually housed in adjustable metabolism crates (1.80 m × 0.60 m) with smooth transparent plastic sides and plastic-covered expanded metal flooring in a temperature controlled room (26 ± 2 °C). Body weight was measured on d 1, 5, 10, 16, daily feed allotment was adjusted accordingly, and pigs were trained to consume the entire meal within 1 h of feeding at 08:00 h. Orts, if any, were measured to accurately determine average daily feed intake (ADFI).

#### Immune challenge

A low dose, repeated LPS challenge, following the modified procedures described by Rakhshandeh and de Lange [8], was chosen to induce an inflammatory response representative of sustained immune system stimulation in the ISS treatment. The challenge consisted of four repeated low-dose injections of *Escherichia coli* LPS serotype O55:B5 (Sigma–Aldrich, St. Louis, MO, USA) for pigs on treatment ISS, or a control injection of sterile saline for pigs in treatments CON and ENZ. The LPS was dissolved in sterile PBS so that an injection of 0.1 mL/kg of BW achieved the desired dosage [13].

A pilot study with 12 pigs was conducted prior to experiment initiation to discern the lowest appropriate initial LPS dose and the subsequent dose increase regimen required to limit LPS tolerance development. Results of the pilot study (not reported herein) indicated that an initial dose of 20 μg LPS/kg of BW with subsequent dose increases of 20%, 30%, and 40% was the regimen that maintained a febrile response (rectal temperature ≥ 40 °C) at all four challenges while minimizing anorexia and vomiting.

During the main experiment, pigs were injected intramuscularly at 10:00 h on d 10, 12, 14, and 16 with either sterile saline or LPS, following the previously described dosing regimen determined from the pilot study. Baseline rectal temperature was measured on d 5 and 8 at 14:00 h and at 4 h post-challenge (14:00 h) on d 10, 12, and 14. Blood samples were then collected on d 8 (pre-challenge) and d 10 (post-challenge) via jugular venipuncture into two 10-mL tubes for EDTA-whole blood and serum. Whole blood samples were placed on ice pending transportation to the laboratory for complete blood count (CBC) analysis with white blood cell (WBC) manual differential. Serum was separated by centrifugation (2,000×*g* for 15 min at 4 °C), collected and divided into three subsamples, and stored at − 80 °C until analyzed.

#### Digestibility

On d 10, pigs received 5 g of ferric oxide as an indigestible marker mixed with 100 g of feed; the remaining allotted feed was offered after the marked feed was consumed. Fecal collection commenced when the marker first appeared in the feces. On d 15, pigs were offered 100 g of marked feed as previously described, and fecal collection terminated when the marker appeared in the feces. Feces were weighed and stored at − 20 °C until further processing. Total urine collection commenced at 08:00 h on d 11 and terminated at 08:00 h on d 16. Urine was collected once daily into jugs containing 10 mL of 6 mol/L HCl. Urine was weighed, thoroughly mixed and subsampled (10% of urine weight), strained through glass wool, and stored at − 20 °C. Urine subsamples were pooled per pig throughout the collection period.

#### Heat production

On d 16, within 30 min of consuming their daily feed allotment, pigs were transferred to open-circuit indirect calorimetry chambers (1.22 m × 0.61 m × 0.91 m metallic box with a glass door on the front side, plastic-covered expanded metal sheet flooring, and a valve at the bottom to collect urine; Columbus Instruments, Columbus, OH, USA) for 34 h of calorimetric measurements. Pigs were randomly assigned to chambers to reduce the possibility of a chamber bias. The first 2 h of HP (08:00–10:00 h), measured prior to pigs receiving the fourth and final challenge of either LPS or saline, were designated as acclimation and not included in HP calculations. Total oxygen consumption (VO_2_) and carbon dioxide production (VCO_2_) were measured every 12 min corresponding to 170 values over 34 h. The first 24 h following feeding was designated as the fed state and the last 12 h as the fasting state [14, 15]. Urine voided during the fed and fasting periods was collected separately and processed as previously described. Personnel movement within the room was minimized during HP measurement to avoid any disturbance of the pigs. The system was validated using the alcohol combustion method described by Aulick et al. [16] and the O_2_ and CO_2_ sensors were calibrated prior to each block of the experiment. The chambers were air-conditioned to maintain a constant temperature (23 ± 1 °C). Pig BW on d 16 was similar across all treatments (14.1 ± 0.3 kg). Heat production was measured in only seven of the ten experimental blocks because of equipment failure during three blocks; therefore, for HP data, *n* = 7.

### Analytical methods

All diet, orts, and fecal samples were dried at 60 °C to a constant weight and were ground to a particle size of 1 mm. Urine samples were thawed, sieved through cotton gauze, and filtered with glass wool. Urine, diet, and fecal samples were analyzed in duplicate for nitrogen (N; method 990.03 [17]; TruMac®; LECO Corp., St. Joseph, MI, USA). An EDTA sample (9.56% N) was used as the standard for calibration and was determined to be (9.55 ± 0.01)% N. Crude protein (CP) was calculated as N × 6.25. Diets were analyzed for mannan (Galactomannan Assay Kit, Megazyme International, Wicklow, Ireland) and β-mannanase concentration (colorimetric determination, Elanco Animal Health, Gaithersburg, MD).

Diet and fecal samples were analyzed in duplicate for dry matter (DM; method 930.15), acid hydrolyzed ether extract (EE; method 2003.06), and starch (Total Starch Assay Kit, Megazyme International, Wicklow, Ireland, method 996.11) using standard methods [17]; and in triplicate for neutral and acid detergent fiber components (NDF [18] and ADF [19], respectively). Hemicellulose was calculated as the difference between NDF and ADF concentrations. Gross energy (GE) was determined using a bomb calorimeter (model 6200; Parr Instrument Co., Moline, IL). Benzoic acid (6,318 kcal GE/kg; Parr Instrument Co.) was used as the standard for calibration and was determined to contain 6,325 ± 6.9 kcal GE/kg. Urine GE was calculated as 192 plus 31 times the concentration of urinary N [20] and multiplied by a factor of 0.239 to convert the unit to kcal.

Pre- and post-challenge whole blood samples and blood smears were analyzed for CBC performed by Manitoba Veterinary Diagnostic Services (Winnipeg, MB, Canada) using Advia 2120i (Siemens Healthcare Diagnostics, Tarrytown, NY, USA) with a manual differential. The main response variables of interest were total cell, RBC, and WBC (mature and immature neutrophils, eosinophils, basophils, lymphocytes, and monocytes) concentrations.

Serum was divided into three subsamples. One set was analyzed for glucose and insulin concentrations by Animal Health Laboratory (University of Guelph, ON, Canada). Glucose concentration was determined on an automated Roche Cobas C501 analyzer (GLUC3 application, Roche Diagnostics, Indianapolis, IN, USA) and insulin concentration was quantified using commercial RIA kits (PI-12 K, EMD Millipore, Billerica, MA, USA). The second serum subset was analyzed for cytokine concentrations (granulocyte macrophage colony-stimulating factor (GM-CSF), tumor necrosis factor alpha (TNFα), Interleukin (IL)-one-alpha (IL-1α), IL-1β, IL-one-receptor antagonist (IL-1ra), IL-2, IL-4, IL-6, IL-8, IL-10, IL-12, and IL-18) by a commercial multiplex assay using laser bead technology (Eve Technologies, Calgary, AB, Canada). The third serum set was analyzed for concentration of APPs haptoglobin and mannose-binding lectin A (MBL) using porcine-specific commercial ELISA kits (Immunology Consultants Laboratory, Inc., Portland, OR, USA; MyBioSource, Inc., San Diego, CA, USA, respectively).

### Calculations

Dry matter, GE, CP, EE, starch, hemicellulose, NDF, and ADF apparent total tract digestibility (ATTD; %) were calculated on a DM basis as [(nutrient intake – nutrient output in feces)/nutrient intake] × 100. Digestible energy (DE) content of the diet was calculated as GE × GE ATTD. Dietary ME was calculated according to the equation of Noblet et al. [21]: ME = DE – [urine GE + (0.4% of DE intake)]. Nitrogen retention (NR) was calculated by the difference between N intake and N excreted in the feces and urine, and protein deposition (PD) was determined as NR × 6.25.

Heat production was calculated from respiratory gas exchanges and urinary N production according to the equation of Brouwer [22]: HP (kcal) = 3.87 × VO_2_ consumed (L) + 1.20 × VCO_2_ produced (L) – 1.43 × urinary N production (g). Methane production was not accounted for, but has been estimated to be very low in growing pigs (< 1% [23]). All HP parameters were normalized to a period of 24 h, expressed as kcal of heat produced per kg of BW^0.60^ [24] and per kg of DM intake (DMI) in order to remove known effects of variations in BW and DMI [25, 26].

Total heat production (HP_total_) was the average HP during the 22 h of post-challenge, fed state measurement. Total fasting heat production (FHP_total_) was the average HP over the 12 h fasted-state. Because the system was not equipped to quantify and separate HP due to physical activity, fasting heat production (FHP) was derived from the 10 lowest HP values over the fasted-state, reflecting energy metabolism due to basal metabolic rate and not associated with feed consumption, digestion, or physical activity [27, 28]. The respiratory quotient (RQ) was calculated as VCO_2_ divided by VO_2_ during the fed (RQ_fed_) and fasting (RQ_fast_) states.

To best estimate components of HP not attributed to the basal metabolic rate, HP values for physical activity and the thermic effect of feeding (TEF) were calculated. Activity heat production (AHP) was estimated utilizing fed-state HP data measured over 10 h post-challenge to represent normal post-feeding daytime behavior. The difference between the average HP over this 10 h period (HP_10_) and the average of the 10 lowest HP values over the same time (HP_low_; representative of sedentary, resting behavior; [27, 28]) was designated as AHP. The TEF was calculated as the difference between HP_10_ and the sum of AHP and FHP, so that any HP in excess of basal metabolism and activity was partitioned toward TEF. Heat increment (HI) was then calculated as the sum of AHP and TEF. Using these data, the efficiency of utilizing ME for maintenance and growth (k_mg_, %) was calculated as (1 – HI / ME intake) × 100 [25, 29]. To address the primary research question of how an immune challenge and β-mannanase supplementation impact maintenance energy requirements, metabolizable energy used for maintenance (ME_m_) was then calculated as FHP × 100/ k_mg_ [25, 29].

Together, dietary energy, N balance, and HP values were utilized to characterize energy use and balance in the pig. Retained energy (RE) was calculated by the difference of ME intake and the total HP during the 24 h fed-state (both pre- and post-challenge) to account for all energy not available for tissue accretion [29]. Energy retained as protein (RE_p_) was calculated from N balance assuming a PD (g) energy value of 5.64 kcal/g [30]. Energy retained as lipid (RE_l_) was calculated as the difference between RE and RE_p_ [30]. Lipid deposition (LD) was then determined from RE_l_ assuming an energy content of 9.49 kcal/g of deposited lipid [22]. Dietary net energy (NE; kcal/kg) was calculated as the sum of RE and FHP divided by DMI [21].

### Statistical analyses

Data were analyzed as a randomized complete block design with pig as the experimental unit. The UNIVARIATE procedure of SAS (Version 9.4, SAS Inst., Cary, NC) was used to verify normality and homogeneity of variances. Statistical outliers (> 3 SD away from the mean) were removed; therefore, one pig from the ISS treatment was removed from HP data because of poor feed intake on the day of HP measurement. Immature neutrophil, eosinophil, and basophil CBC data were log transformed to achieve a normal distribution.

The main effects of dietary treatment and block were analyzed using the MIXED procedure of SAS. The staggered block experimental design resulted in variations in time and body weight among blocks. This variation was expected and resulted in statistical detection of block as a significant main effect in most response variables. Therefore, block remained in the statistical model, but block *P*-values are not reported herein.

Differences among treatments were determined using ANOVA and means were separated using the least square means statement and the PDIFF option. Immune and rectal temperature data were analyzed as repeated measures and covariance structures resulting in the lowest AIC values for each variable were applied. To further evaluate β-mannanase effects on immune parameters pre-challenge, contrasts comparing CON versus ENZ and ISS values were generated using the contrast statement of the MIXED procedure. Differences were considered significant if *P* was ≤0.05 and a trend if *P* was > 0.05 and ≤ 0.10.

## Results

### Immune response parameters

#### Immune system stimulation effects

**Fig. 1:**
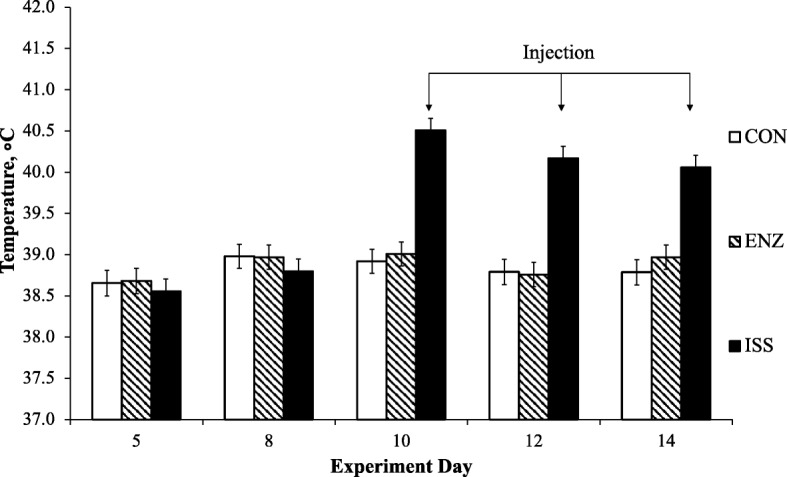
Effect of treatment on young weaned pig (*n* = 10 per treatment) rectal temperature (°C). Control treatment (CON) = pigs fed basal diet (0.0% β-mannanase) with saline injection. Enzyme treatment (ENZ) = pigs fed enzyme diet (0.056% β-mannanase) with saline injection. Immune system stimulation treatment (ISS) = pigs fed enzyme diet (0.056% β-mannanase) with LPS (*Escherichia coli* serotype O55:B5) injection. The arrows indicate days on which either a saline (CON and ENZ) or LPS (ISS) injection were administered at 10:00 h. Rectal temperatures were measured 4 h post-challenge. Data points on d 5 and 8 represent average baseline pre-challenge temperature, and d 10, 12, and 14 represent post-challenge temperatures. Treatment by day interaction *P* < 0.0001; day *P* < 0.0001; treatment *P* < 0.0001; block *P* = 0.0015

Pigs on the ISS treatment exhibited minimal vomiting, diarrhea and signs of lethargy and hyperventilation after the first and to a lesser extent, the second LPS injection. After the third and fourth challenges, ISS pigs continued to demonstrate signs of lethargy and hyperventilation, but vomiting and diarrhea were not observed. No pigs died after any injection. The immune stimulation model successfully induced a sustained febrile response (rectal temperature ≥ 40 °C) in ISS pigs on d 10, 12, and 14 (treatment by day interaction *P* < 0.0001; Fig. 1). Pigs in CON and ENZ treatments maintained normal rectal temperatures (38.85 °C ± 0.15) throughout the experiment.

**Fig. 2:**
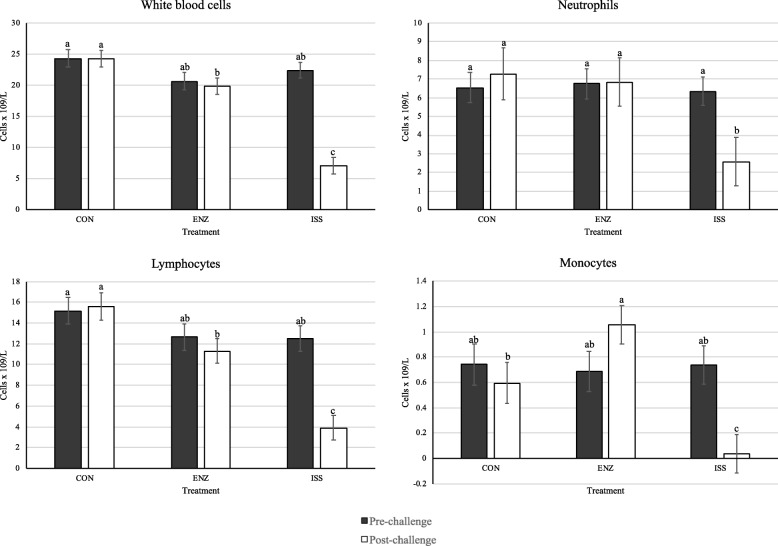
Effect of treatment on complete blood count before (d 8) and after (d 10) challenge. Serum was collected at 14:00 h each day (4 h post- challenge). Control treatment (CON) = pigs fed basal diet (0.0% β-mannanase) with saline injection. Enzyme treatment (ENZ) = pigs fed enzyme diet (0.056% β-mannanase) with saline injection. Immune system stimulation treatment (ISS) = pigs fed enzyme diet (0.056% β-mannanase) with LPS (*Escherichia coli* serotype O55:B5) injection. *n* = 10 per treatment. ^a,b^Within a graph, bars without a common superscript differ, *P* < 0.05

There was a significant interaction between the effects of time (pre- or post-challenge) and treatment on WBC, mature neutrophil, lymphocyte, and monocyte counts and a trend for an interaction on RBC count (Additional file 2: Table S2). In all four variables, treatments had similar cell counts at the pre-challenge time point (*P* ≥ 0.10). Post-challenge, immune stimulation by LPS decreased WBC, mature neutrophil, lymphocyte, and monocyte counts compared to CON and ENZ (*P* ≤ 0.05; Fig. 2). There were no differences among treatments or time periods for total cell, immature neutrophil, eosinophil, or basophil counts (*P* > 0.10; Additional file 2: Table S2).

Glucose, insulin, haptoglobin, and MBL serum concentrations were not affected by the interaction of time and treatment (*P* > 0.10), but concentrations were higher pre-challenge compared to post-challenge for glucose, haptoglobin, and MBL (Additional file 3: Table S3).

**Fig. 3:**
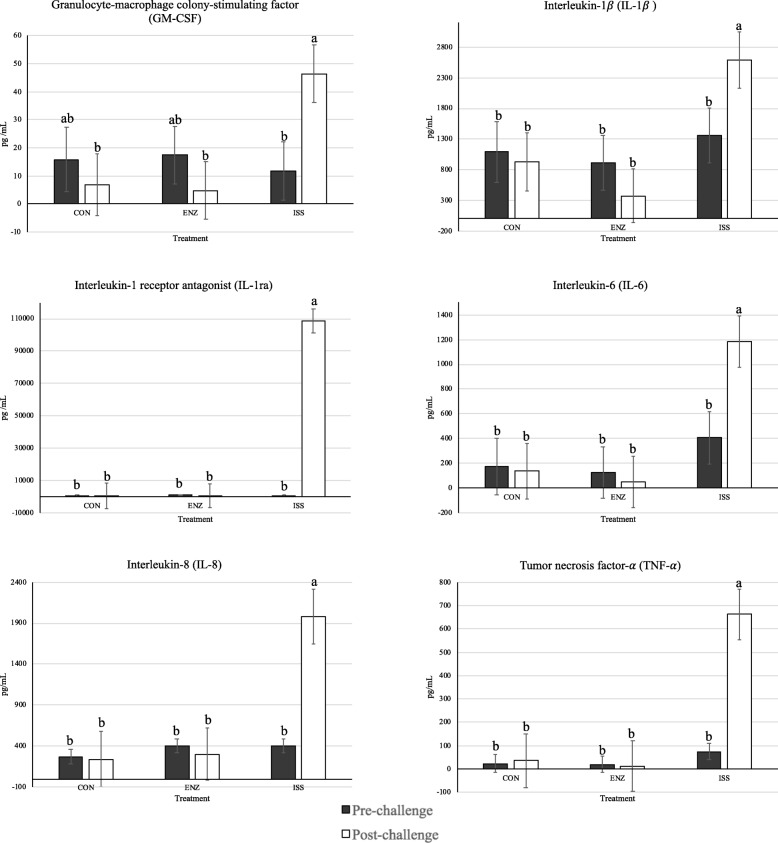
Effect of treatment on serum cytokine concentrations before (d 8) and after (d 10) challenge. Serum was collected at 14:00 h each day (4 h post- challenge). Control treatment (CON) = pigs fed basal diet (0.0% β-mannanase) with saline injection. Enzyme treatment (ENZ) = pigs fed enzyme diet (0.056% β-mannanase) with saline injection. Immune system stimulation treatment (ISS) = pigs fed enzyme diet (0.056% β-mannanase) with LPS (*Escherichia coli* serotype O55:B5) injection. *n* = 10 per treatment. ^a,b^Within a graph, bars without a common superscript differ, *P* < 0.05

Lipopolysaccharide challenge increased IL-1β, IL-1ra, IL-6, IL-8, and TNFα concentrations post-challenge compared to ISS pre-challenge and both pre- and post-challenge concentrations in CON and ISS (Fig. 3). All other cytokines were not significantly impacted by the interaction or main effects of time and treatment (*P* > 0.10; Additional file 3: Table S3). Interferon-gamma was not detected in any of the samples. Serum GM-CSF concentrations were not different (*P* > 0.10) among CON and ENZ pre-and post-challenge and ISS pre-challenge, while ISS post-challenge GM-CSF concentration was increased compared to the ISS pre-challenge value and CON and ENZ post-challenge values (*P* ≤ 0.015; Fig. 3).

#### β-mannanase effects

Contrasts comparing immune cell dynamics of pigs fed either the control or β-mannanase diet prior to the first challenge on d 10 detected no differences in CBC values (*P* ≥ 0.10; Table 3). Similarly, serum glucose, insulin, MBL, and cytokine concentrations (except IL-1α) did not differ because of β-mannanase supplementation (*P* ≥ 0.230; Table 4). Serum haptoglobin and IL-1α concentrations were decreased in diets supplemented with β-mannanase (*P* ≤ 0.05; Table 4).

**Table 3:** Complete blood count values in young weaned pigs fed a diet with or without β-mannanase^a,b^

Treatment	Control diet^c^		β-mannanase diet^d^		Contrast *P*-value
	Estimate	SEM	Estimate	SEM	
Cell type count, cells × 10^9^/L^e^					
Total cells	468.6	34.9	432.4	23.8	0.405
WBC	24.50	1.43	21.51	0.98	0.104
Neut	6.47	0.94	6.83	0.64	0.758
Bands	0.200	0.050	0.266	0.034	0.504
Eos	0.202	0.099	0.300	0.068	0.599
Baso	0.048	0.061	0.100	0.042	0.451
Lymph	15.35	1.46	12.46	1.00	0.124
Mono	0.727	0.181	0.709	0.123	0.934
RBC	7.44	0.14	7.55	0.09	0.547

^a^Blood was collected on d 8 of the experiment 6 h post-feeding, prior to the immune challenge beginning on d 10

^b^*n* = 10 pigs per treatment

^c^Control diet was a corn, soybean mean, and soy hulls based diet containing 1.33% β-mannans, and did not contain β-mannanase enzyme. Pigs on the control (CON) treatment were fed the control diet and estimates are representative of the CON treatment only

^d^Enzyme diet was the control diet supplemented with 0.056% β-mannanase (Hemicell™ HT-D, Elanco Animal Health, Guelph, ON, Canada; endo-1,4-β-mannanase (160 × 10^6^ units/kg) from *Paenibacillus alvei*). Pigs on the enzyme (ENZ) and immune system stimulation (ISS) treatments were fed the enzyme diet. Estimates are representative of the ENZ and ISS treatments prior to immune stimulation

^e^Basophils (Baso); eosinophils (Eos); immature neutrophils (Bands); lymphocytes (Lymph); mature neutrophils (Neut); monocytes (Mono); white blood cells (WBC)

**Table 4:** Effect of β-mannanase on pig serum glucose, insulin, acute phase protein, and cytokine concentrations^a,b^

Treatment	Control diet^c^		Enzyme diet^d^		Contrast *P*-value
	Estimate	SEM	Estimate	SEM	
Glucose, mmol/L	7.70	0.46	7.41	0.25	0.585
Insulin, pmol/L	88.92	10.99	83.35	6.06	0.664
Insulin:Glucose	11.56	1.21	11.08	0.67	0.734
Acute phase protein, mg/mL					
Haptoglobin	1.57	0.22	1.02	0.14	0.050
MBL^e^	125.4	8.6	117.5	5.2	0.445
Cytokine, pg/mL^f^					
GM-CSF	17.01	13.01	14.56	7.85	0.874
IL-1α	35.01	6.81	7.50	4.27	0.004
IL-1β	1064	598	1138	361	0.917
IL-1ra	428.6	257.5	673.7	161.5	0.435
IL-2	328.4	157.6	228.8	95.0	0.596
IL-4	995.5	545.6	711.6	329.0	0.662
IL-6	190.2	61.5	98.72	38.59	0.230
IL-8	263.1	95.4	402.2	57.5	0.230
IL-10	499.4	159.1	332.9	95.9	0.383
IL-12	1570	173	1730	104	0.439
IL-18	1994	546	1380	329	0.350
TNFα	24.29	39.90	52.30	25.02	0.563

^a^Blood was collected on d 8 of the experiment 6 h post-feeding, prior to the immune challenge beginning on d 10

^b^*n* = 10 pigs per treatment

^c^Control diet was a corn, soybean mean, and soy hulls based diet containing 1.33% β-mannans, and did not contain β-mannanase enzyme. Pigs on the control (CON) treatment were fed the control diet and estimates are representative of the CON treatment only

^d^Enzyme diet was the control diet supplemented with 0.056% β-mannanase (Hemicell™ HT-D, Elanco Animal Health, Guelph, ON, Canada; endo-1,4-β-mannanase (160 × 10^6^ units/kg) from *Paenibacillus alvei*). Pigs on the enzyme (ENZ) and immune system stimulation (ISS) treatments were fed the enzyme diet. Estimates are representative of the ENZ and ISS treatments prior to immune stimulation

^e^Mannose binding lectin A (MBL)

^f^Granulocyte-macrophage colony-stimulating factor (GM-CSF); interleukin-1α (IL-1α); interleukin-1β (IL-1β); interleukin-1 receptor antagonist (IL-1ra); interleukin-2 (IL-2); interleukin-4 (IL-4); interleukin-6 (IL-6); interleukin-8 (IL-8); interleukin-10 (IL-10); interleukin-12 (IL-12); interleukin-18 (IL-18); tumor necrosis factor alpha (TNFα)

### Pig growth performance, nitrogen balance, and diet digestibility

Average initial BW was 10.27 ± 0.15 kg, d 16 average BW was 15.12 ± 0.27 kg, and BW did not differ among treatments at either time point (*P* ≥ 0.471). Average daily gain (ADG) over the entire 16-d experiment was not different among treatments (*P* = 0.13; Table 5), but ISS ADG during the immune challenge (d 10–16) was less than CON and ENZ gain (*P* = 0.010; Table 7).

**Table 5:** Growth performance and nitrogen balance in pigs on control, enzyme, or immune system stimulation treatment^c^

Item	CON^d^	ENZ^e^	ISS^f^	SEM	Treatment *P*-value
Body Weight, kg					
d 0	10.23	10.21	10.38	0.15	0.651
d 16	15.25	15.26	14.86	0.27	0.471
ADG d 1–16, g/d	313.9	316.0	279.7	13.6	0.128
Nitrogen (N) balance, g/d					
Intake	21.02^a^	20.93^a^	17.38^b^	0.95	0.021
Excreted	6.21	7.08	6.24	0.45	0.318
In feces	2.62^a^	2.83^a^	2.27^b^	0.11	0.007
In urine	3.59	4.25	3.97	0.47	0.624
Retained	14.81^a^	13.85^ab^	11.14^b^	0.99	0.045
% of excreted N in feces	41.31	41.21	38.42	3.29	0.778
% of excreted N in urine	58.69	58.79	61.58	3.29	0.778
Nitrogen balance, % of intake					
Excreted in feces	12.41	13.54	13.23	0.44	0.212
Excreted in urine	17.38	21.40	24.56	4.14	0.495

^a,b^Within a row, treatment means without a common superscript differ, *P* < 0.05

^c^*n* = 10 pigs per treatment

^d^Control treatment (CON) = pigs fed basal diet (0.0% β-mannanase) with saline injection

^e^Enzyme treatment (ENZ) = pigs fed enzyme diet (0.056% β-mannanase) with saline injection

^f^Immune system stimulation treatment (ISS) = pigs fed enzyme diet (0.056% β-mannanase) with LPS (*Escherichia coli* serotype O55:B5) injection

Immune system simulation numerically decreased ADFI and thus N intake compared to CON and ENZ (*P* = 0.021), resulting in decreased fecal N excretion on a g per d basis (*P* = 0.007). Urine N excretion during the challenge period was similar among treatments (*P* = 0.045), but retained N in the ISS treatment was less than that of CON, with ENZ being intermediate (*P* = 0.045; Table 5). Partitioning of excreted N to either the feces or urine was not different among treatment (*P* = 0.78). When N excretion was expressed as a percent of N intake, the previously observed significant treatment effect on fecal N excretion was no longer evident (Table 5). There were no differences among treatments in ATTD of any analyzed nutrient (*P* ≥ 0.120; Table 6) and all ATTD coefficients were within normal ranges for 10 to 15 kg pigs.

**Table 6:** Apparent total tract digestibility in pigs on the control, enzyme, or immune system stimulation treatment^a^

Item	CON^b^	ENZ^c^	ISS^d^	SEM	Treatment *P*-value
ATTD^e^, %					
DM	88.05	87.36	87.66	0.33	0.356
GE	87.35	86.73	86.86	0.33	0.418
CP	87.59	86.47	86.77	0.44	0.212
EE^f^	70.41	69.62	66.85	1.18	0.122
Starch	99.41	99.50	99.56	0.10	0.565
NDF	68.81	66.11	70.23	1.55	0.178
ADF	71.65	68.04	74.13	2.26	0.175
Hemicellulose^g^	65.00	63.51	64.99	0.92	0.429

^a^*n* = 10 pigs per treatment

^b^Control treatment (CON) = pigs fed basal diet (0.0% β-mannanase) with saline injection

^c^Enzyme treatment (ENZ) = pigs fed enzyme diet (0.056% β-mannanase) with saline injection

^d^Immune system stimulation treatment (ISS) = pigs fed enzyme diet (0.056% β-mannanase) with LPS (*Escherichia coli* serotype O55:B5) injection

^e^ATTD, % = [(nutrient intake (kg) – fecal nutrient output (kg)) / nutrient intake (kg)] × 100

^f^Acid hydrolyzed ether extract

^g^Hemicellulose = NDF - ADF

### Heat production, maintenance energy requirements, and energy retention

Day 16 ME intake was similar among treatments (759.4 ± 37.7 kcal/kg BW^0.60^/kg DMI/d; *P* = 0.92). Immune system stimulation increased fed state HP_total_ compared to CON and ENZ (*P* = 0.040; Table 7). In the fasting state, neither immune stimulation nor β-mannanase supplementation affected FHP_total_ or FHP compared to control (*P* ≥ 0.135). Treatment did not affect RQ in the fed and fasting states (*P* ≥ 0.23; Table 7).

**Table 7:** Effect of treatment on energy balance, respiratory quotient, maintenance energy requirements, and nutrient deposition^c^

	Treatment				Treatment *P*-value
Item	CON^d^	ENZ^e^	ISS^f^	SEM	
Day 16 BW, kg	14.37	14.19	13.77	0.26	0.313
Day 16 DMI, kg	0.51	0.51	0.46	0.03	0.348
Energy balance, kcal/kg BW^0.60^/kg DMI/d					
ME intake	771.3	755.9	751.1	37.7	0.924
Heat production^g^					
HP_total_	278.8^b^	274.9^b^	333.0^a^	14.9	0.040
FHP_total_	287.8	276.0	324.3	17.1	0.178
FHP	207.8	206.6	243.3	12.9	0.135
Retained energy^h^					
As protein	197.5	173.6	191.0	18.3	0.627
As lipid	291.4^a^	302.9^a^	227.7^b^	19.2	0.046
Total	488.9	476.5	418.7	32.0	0.318
k_mg_, %	87.07	86.44	83.01	1.34	0.130
Estimated ME_m_	239.0^b^	239.5^b^	295.5^a^	15.3	0.045
Retained energy, % of ME intake					
As protein	25.68	23.15	24.99	1.27	0.354
As lipid	37.77^a^	40.07^a^	29.81^b^	2.02	0.013
Total	63.44^a^	63.22^a^	54.80^b^	2.18	0.033
Respiratory quotient					
Fed state	0.92	0.90	0.88	0.01	0.225
Fasting state	0.74	0.73	0.73	0.01	0.381
Nutrient deposition^i^, g/d					
As protein	87.74	78.55	69.80	5.86	0.150
As lipid	76.22^a^	79.43^a^	55.45^b^	6.21	0.047
ADG d 10–16, g/d	447.1^a^	404.8^a^	330.7^b^	21.3	0.010

^a,b^Within a row, treatment means without a common superscript differ, *P* < 0.05

^c^*n* = 7 pigs per treatment (CON and ENZ) and 6 pigs per treatment (ISS)

^d^Control treatment (CON) = pigs fed basal diet (0.0% β-mannanase) with saline injection

^e^Enzyme treatment (ENZ) = pigs fed enzyme diet (0.056% β-mannanase) with saline injection

^f^Immune system stimulation treatment (ISS) = pigs fed enzyme diet (0.056% β-mannanase) with LPS (*Escherichia coli* serotype O55:B5) injection

^g^Heat production (HP) = (3.87 × O_2_ consumption (L) + 1.20 × CO_2_ production (L) – 1.43 × urinary N)/BW^0.60^ (kg) [22]; Total HP (HP_total_) = avg. HP over 22 h fed state, post- challenge; Total fasting HP (FHP_total_) = avg. HP over 12 h fasted state; Fasting HP (FHP) = avg. of 10 lowest HP values over the 12 h fasted state [27, 28]; HP_10_ = avg. HP over 10 h post-challenge (10:00 h – 20:00 h), fed state; HP_low_ = avg. of 10 lowest HP values over 10 h post-challenge (10:00 h – 20:00 h), fed state; Activity HP (AHP) = HP_10_ - HP_low_; Thermic effect of feeding (TEF) = HP_10_ - AHP – FHP; Heat increment (HI) = AHP + TEF; ME efficiency for maintenance and growth (k_mg_) = (1 – HI) × 100 [25]; ME used for maintenance (ME_m_) = FHP × 100/k_mg_ [25]

^h^Retained energy (RE) = ME intake – total fed-state HP, pre-and post-challenge [29]; RE as protein (RE_p_) = [PD (g) × 5.66 (kcal/g)]/ BW^0.60^/DMI [30]; RE as lipid (RE_l_) = RE - RE_p_ [30]

^i^Protein deposition = nitrogen retention (g) × 6.25; Lipid deposition = RE_f_ (kcal) / 9.49 (kcal/g) [30]

Immune system stimulation increased ME_m_ (kcal/kg BW^0.60^/kg DMI/d) compared to CON or ENZ pigs (*P* = 0.045), but k_mg_ among treatments did not differ (*P* = 0.13; Table 7). When ME_m_ was expressed as kcal/d, the significant treatment effect was no longer detected (*P* = 0.90). Beta-mannanase supplementation did not change ME_m_ relative to CON whether expressed as kcal/kg BW^0.60^/kg DMI/d (*P* = 0.98), kcal/kg BW/d (*P* = 0.72), or kcal/d (*P* = 0.77).

Absorbed energy not lost via urine, gases, heat increment, activity and TEF, or maintenance, is retained as either protein or lipid. Immune system stimulation decreased RE_l_ compared to CON and ENZ (*P* = 0.046) but RE_p_ and total RE were not different among treatments (*P* > 0.32) when expressed as kcal/kg BW^0.60^/kg DMI/d (Table 7). When RE was expressed as a proportion of ME intake, similar treatment effects were observed for RE_l_ and RE_p_, but a significant decrease in total RE was detected due to ISS (*P* = 0.033; Table 7). As less energy was retained as lipid, LD was decreased in the ISS treatment compared to CON and ENZ (*P* = 0.047) while no differences were observed in PD (*P* = 0.15; Table 7).

### Dietary energy values and efficiency

The ENZ and ISS treatments tended to decrease diet DE and ME values relative to CON (*P* ≤ 0.052; Table 8). Neither ISS nor β-mannanase supplementation (ENZ treatment) affected dietary NE value (*P* = 0.75) or ME and NE efficiency (*P* ≥ 0.46).

**Table 8:** Dietary energy values and efficiency in pigs on control, enzyme, or immune system stimulation treatment^a^

	Treatment				Treatment *P*-value
Item	CON^b^	ENZ^c^	ISS^d^	SEM	
Dietary energy value^e^, Mcal/kg DM					
GE	4.65	4.59	4.59		
DE	4.07	3.99	4.00	0.02	0.051
ME	3.96	3.86	3.89	0.03	0.052
NE	3.29	3.30	3.11	0.19	0.748
ME/DE efficiency, %	97.31	96.92	97.35	0.37	0.457
NE/ME efficiency, %	83.09	85.31	80.03	4.42	0.701

^a^*n* = 7 pigs per treatment (CON and ENZ) and 6 pigs per treatment (ISS)

^b^Control treatment (CON) = pigs fed basal diet (0.0% β-mannanase) with saline injection

^c^Enzyme treatment (ENZ) = pigs fed enzyme diet (0.056% β-mannanase) with saline injection

^d^Immune system stimulation treatment (ISS) = pigs fed enzyme diet (0.056% β-mannanase) with LPS (*Escherichia coli* serotype O55:B5) injection

^e^Gross energy (GE) analyzed via bomb calorimetry; digestible energy (DE) = GE apparent total tract digestibility coefficient × diet GE; metabolizable energy (ME) = DE – (urinary energy + 0.4% of DE intake); net energy (NE) = (retained energy + fasting heat production)/DMI

## Discussion

During an immune challenge, pro-inflammatory cytokines initiate a shift in nutrient partitioning away from tissue growth to support activation and maintenance of an immune response [1, 11, 31]. The results of this experiment clearly demonstrated that a systemic inflammatory response to LPS occurred, verified by increased concentrations of pro-inflammatory cytokines and elevated body temperature. To our knowledge, these data are the first to directly relate decreased ADG to increased ME_m_ independent of changes in feed intake during an immune response. Additionally, this experiment provides novel data on β-mannanase supplementation effects on immune parameters and energy balance in pigs.

### β-mannanase

As a constituent of hemicellulose, β-mannan is not digested by mammalian endogenous enzymes [32]. Thus, intact β-mannans are available to bind carbohydrate recognition domains of pattern recognition receptors on innate immune cells surveying the intestinal epithelium for potential pathogens [3, 33]. In this way, β-mannans are hypothesized to be capable of stimulating innate immune cells resulting in a nonproductive, energy draining immune response [4–6].

Commonly, only growth and feed efficiency responses have been measured from β-mannanase and reported in the animal nutrition literature. Reduced feed efficiency and ADG have been reported with increasing dietary β-mannan concentrations [34]. Therefore, there is interest in β-mannanase supplementation to alleviate these negative effects by enzymatic hydrolysis of β-mannan polysaccharides. The FIIR was alleviated through β-mannanase supplementation in poultry [6, 35]; however, β-mannanase supplementation responses in swine have been inconsistent. This experiment demonstrated no β-mannanase effect on the ATTD of DM, GE, CP, EE, or hemicellulose. Growth performance responses are similarly inconsistent with positive results in some studies [36–38] but no β-mannanase effect in others [39–42]. In this experiment, ENZ did not improve ADG, protein, or lipid deposition.

Dietary β-mannans are proposed to stimulate the innate immune system through direct interactions with the carbohydrate binding domains of mannose recognition receptors such as the membrane bound mannose receptor and secreted MBL. Therefore, serum MBL concentrations were measured to determine if β-mannanase supplementation decreased circulating MBL, theoretically by removing the substrate for activation and synthesis. Serum MBL concentrations were not affected by β-mannanase. This may indicate that the β-mannan concentration in the intestinal lumen was not high enough to either interact with MBL, MBL-dietary β-mannan interaction was not affected by β-mannanase supplementation, or this interaction is not a mechanism through which β-mannans are sensed by the innate immune system.

Two significant differences in serum parameters were detected when contrasts were applied to compare pre-challenge values between control pigs (no β-mannanase, CON treatment) and β-mannanase supplemented pigs (ENZ and ISS treatments). Beta-mannanase supplementation decreased serum haptoglobin and IL-1α concentrations. In poultry, decreased haptoglobin has been proposed as evidence of immune stress alleviation due to β-mannanase supplementation [6]. However, this response occurred in conjunction with growth performance and feed efficiency improvements which were not observed in this study. Beta-mannanase effects on IL-1α concentrations have not been previously reported. Interleukin-1-alpha can be involved in inflammation initiation, but the relationship between serum concentration and magnitude of immune challenge is not as clear as the implication of its counterpart, IL-1β on systemic inflammation [43]. Interleukin-1-beta concentrations were not affected by β-mannanase supplementation in this study.

In total, decreased serum IL-1α and haptoglobin concentrations are not strong enough evidence of an alleviated systemic FIIR when taken in context with the lack of all other measured inflammatory-type variables. Importantly, no differences were observed in HP, ME_m_, and growth performance. It is possible that a localized response may have occurred at the intestinal level yet went undetected systemically. However, if this occurred, whole body nutrient and energy partitioning were still unaffected. The hypothesis that β-mannanase supplementation would decrease ME_m_ was not supported. Pigs fed diets supplemented with β-mannanase had similar WBC counts, cytokine concentrations, nutrient digestibility, ADG, N and energy balance, PD, LD, and ME_m_ compared to CON pigs.

### Immune stimulation

Innate immune stimulation was successfully induced in pigs using sequential, increasing doses of *E. coli* LPS. Elevated rectal temperature, increased pro-inflammatory cytokine concentrations, and altered nutrient and energy partitioning are all hallmarks of a chronic immune challenge [1] and were observed in ISS pigs in this study. One limitation of this study was the number of calorimetry chambers available which limited the experiment to a total of three treatments. Due to this limitation, we were unable to evaluate the interaction of β-mannanase supplementation with LPS immune stimulation. Thus, interpretation of ISS effects has been made in comparison to the ENZ treatment. However, as discussed above, there were no differences between the CON and ENZ treatments in nutrient digestibility, ADG, or N and energy balance. The major finding of this research indicates that the innate immune challenge increased young pig maintenance energy requirements by 23.3% which translated into a 18.3% decrease in ADG.

Unique to this study, decreased ADG could be attributed primarily toward increased ME_m_ in ISS pigs as opposed to decreased feed intake or effects on nutrient digestibility. Anorexia is a well-established response to systemic immune stimulation [2, 9, 44] induced by pro-inflammatory cytokine actions (especially IL-1β) in the brain and modulation of metabolism and hormone release [45]. In this study, a numerical but not statistically significant decrease in ADFI was observed in ISS pigs during the challenge period even though IL-1β increased. It is likely that a stronger ADFI decrease was not observed as a consequence of challenging the pigs 2 h post-feeding and limit feeding to 2.5 times maintenance energy requirements [12]. This feeding level was designed to achieve similar ADFI for pigs on all treatments because of the known effect of previous feeding level on HP [25]. To further ensure HP results were separated from feed intake and BW effects, all energy balance calculations were conducted on a kcal/BW^0.60^/DMI/d basis. Just as feed intake did not influence the observed decrease in ADG of ISS pigs, nutrient digestibility was not different across treatments. This is in agreement with other studies reporting ATTD during a chronic LPS challenge [46, 47].

#### Febrile response

Before the challenge period, rectal temperatures and blood immune parameters in ISS pigs were not different from those on the CON and ENZ treatments. This confirmed that prior to the challenge all pigs were in good health and of similar immune status. Therefore, any subsequent differences during the challenge were attributed to LPS immune stimulation. Elevated rectal temperatures (> 40 °C) post-challenge on d 10, 12, and 14 indicated a febrile response in ISS pigs.

Fever is energetically expensive with increased caloric requirement estimates ranging from 7 to 15% for each 1 °C increase in body temperature [48]. Utilizing the average rectal temperature of CON and ENZ pigs and the post-challenge temperature of ISS pigs on day 14, an increase of 1.2 °C resulted in a 23.6% increase in ME_m_ caloric requirements. This value is higher than the previously described range and may indicate that the majority, but not all of the increase in maintenance caloric requirement is to support the febrile response. The remainder may be partially explained by an increase in immune cell glucose requirements [11].

#### Cytokines

Key pro-inflammatory cytokines include TNFα, IL-6, and IL-1β [49] and ISS pigs had increased serum concentrations of all three after the first LPS challenge. Pro-inflammatory cytokines shift metabolism away from anabolic processes toward a more catabolic state to generate AAs and energy necessary to support fever, increase immune cell proliferation, and APP synthesis [50, 51]. In this study, the pro-inflammatory cytokine profile of ISS pigs clearly shifted metabolism toward a lipolytic state and this resulted in significantly less energy retained as lipid and decreased lipid deposition compared to non-immunologically challenged pigs.

#### Complete blood count

Immune stimulation decreased WBC counts, specifically neutrophils, lymphocytes, and monocytes. This is similar to other instances of leukopenia observed due to LPS administration [11, 52]. However, WBC distribution drastically changed following LPS administration and circulating concentrations are dependent upon the time of sampling relative to immune challenge [52, 53]. Thus, variable responses in WBC counts have been reported due to LPS immune stimulation. Rakhshandeh and de Lang observed 1.6 times greater WBC [8] in one study, but in a second, WBC count decreased by 9% [54]. At the time of sampling in this study, leukocyte extravasation into the LPS injection site and into immunologically important tissues likely explains the observed leukopenia.

#### Acute phase proteins

In addition to increased pro-inflammatory cytokine production and leukocyte migration that occur during infection, the acute phase response typically includes increased APP synthesis by the liver. However, in this study, ISS APP concentrations did not differ compared to CON. This was an unexpected result because LPS has been demonstrated to increase APPs such as haptoglobin [8, 46] and C-reactive protein [55] in pigs. A less responsive APP, MBL has been demonstrated to attenuate LPS-induced pro-inflammatory cytokine production [56] and inhibit T-lymphocyte activation [57]. However, in this study it did not appear that LPS induced greater MBL or haptoglobin production.

Although a MBL response was not necessarily expected, a haptoglobin response was. Haptoglobin is a primary APP in pigs and is synthesized in the liver when activated by IL-6 and to a lesser extent IL-1 [58], both of which were significantly elevated in ISS pigs post-challenge. Similar to our results, Koopmans et al. [55] discussed unpublished data which showed no LPS effect on haptoglobin concentrations even though there were clear increases in plasma cortisol, TNFα, IL-6, and C-reactive protein over a 24-h period after LPS challenge. One possible explanation for a lack of haptoglobin response could be time related. Serum samples in this study were collected 4 h after the first challenge and haptoglobin may be a better indicator of chronic inflammation [59].

#### Nitrogen balance

Disease is associated with decreased growth performance and changes in nutrient partitioning. Often, N metabolism is affected because of increasing AA requirements for immune cell proliferation and APP synthesis [51]. In this study, only numerical decreases in protein deposition were measured in ISS pigs compared to CON and ENZ. If protein catabolism had increased to provide AAs for APPs, an increase in urinary N would have been expected because APPs have a distinctly different AA profile than skeletal muscle [50, 60]. However, due to the high dietary CP concentration, it is possible that these excessive dietary amino acids may have provided the additional amino acids required for APP synthesis and prevented the typically observed increase in skeletal muscle protein catabolism.

#### Energy balance

Disease is well known to be detrimental to pig efficiency and productivity. A considerable amount of research has focused on products to mitigate the drop in performance [13, 61] or prevent initial disease onset [62]. Yet few studies have evaluated the energetic cost of an immune challenge in order to generate more effective dietary interventions. In this study, total HP increased by 21.1% in ISS pigs compared to the ENZ treatment.

Campos et al. [46] also evaluated HP components during an immune response and reported significant decreases in ADFI leading to decreased TEF compared to baseline values. In this study DMI did not differ, potential feed intake effects on TEF were removed by interpreting the data after normalizing to a constant feed intake, and TEF values were not affected by ISS. Therefore, both experiments indicate that a chronic inflammatory response did not increase HP through increased TEF. This is supported by the lack of treatment differences in diet digestibility and further supports our supposition that the impact of immune stimulation on energy balance in this study is not through influences on diet digestion or nutrient uptake.

However, it is clear that energy partitioning between maintenance and growth was affected by ISS. A 23.3% increase in ME_m_ was detected due to ISS. As caloric requirements for maintenance increased to support the immune system, less dietary energy was retained for growth. This manifested as less RE_l_ resulting in a 30.2% decrease in lipid deposition.

Previous studies across all species have related increased caloric requirements with fever [48, 63], but few have directly related a chronic immune challenge with increased ME_m_. In vitro studies with isolated mitochondria from rats stimulated with TNFα or IL-1 showed up to 30% increases in respiration rate [64]. Demas et al. [63] reported that mice injected with a mild antigen had limited immune activation that resulted in significantly more O_2_ consumption than control mice injected with saline. Interleukin-six infusions in humans increased resting metabolic rates by 25% [65].

In pigs, the direct relationship between immune stimulation and increased energy requirements has not previously been demonstrated. Some studies reported that immune system stimulation did not impact growth, efficiency, or energy balance measurements [66, 67] However, Moon et al. [66] reported fibroblast formation at the injection site which encapsulated the immunogen and prevented systemic delivery. Williams et al. [67] used the comparative slaughter technique and reported no differences in the energetic costs of maintenance, PD, and LD between pigs raised in environments encouraging high or low chronic immune activation.

Conversely, Labussière et al. [68] and Campos et al. [46] reported decreased HP in pigs during inflammatory challenges. Labussière et al. [68] administered a single injection of complete Freund’s adjuvant to young weaned pigs but did not measured HP until the day after challenge and only re-entered the calorimetry chamber after visual recovery [68]; and this likely biased the response. Campos et al. [46] reported a 14% decrease in total HP (kcal/BW^0.60^/d) in response to a repeated LPS challenge in growing pigs even though typical inflammatory-type and febrile responses were observed. Decreased HP was mainly attributed to lower TEF which reflected the effect of feed intake depression on HP. According to the relationship reported by Labussière et al. [25], lower ADFI should have decreased ME_m_ by 24 kcal/BW^0.60^/d. Because this drop in ME_m_ did not occur, the authors reasoned that the immune stimulation did in fact increase ME_m_ relative to baseline [46]. This supports our experimental model of limit feeding to encourage similar feed intake and to evaluate energy balance on a kcal/BW^0.60^/DMI/d basis. Feed intake clearly influences and can bias HP results and interpretations.

Interpretation of our results in context with the previously discussed reports suggests that an inflammatory response does increase ME_m_ relative to healthy control animals, but in some experiments this response may be masked by decreased HP related to decreased feed intake. This may mean that during an immune response the total caloric requirement may not drastically change because of decreased feed intake, but how those calories are partitioned does change; and this results in growth and feed efficiency depressions commonly observed during disease challenges.

These results supported our hypothesis that energy partitioning shifts to allocate more energy for initiation and maintenance of immune functions and less toward nutrient deposition. Other research would support changes in N metabolism [46, 67, 69] whereas our data suggest that less energy was allocated for LD. Both result in decreased ADG and efficiency losses in pork production, yet these effects are generally given little consideration in commercial swine feeding practices.

## Conclusions

This experiment provides novel data on β-mannanase supplementation effects on immune parameters and energy balance in pigs. Beta-mannanase supplementation did not benefit immune status, nutrient digestibility, growth performance, energy balance, or ME_m_ in young pigs fed a corn, soybean meal, and soybean hulls-based diet. More research is needed to determine how β-mannanase functions in pigs and in which environments and diets it might be effective. These novel data directly relate decreased ADG to increased ME_m_ independent of changes in feed intake in immune challenged pigs. An innate immune challenge increased proinflammatory cytokine concentrations which induced a febrile response and elevated HP and ME_m_ by 23.3%. Increased energy partitioning toward the immune response limited LD by 30.2% leading to a 18.3% decrease in ADG during the immune challenge. These data expand upon the available literature to describe the magnitude of increase in ME_m_ in immune challenged pigs relative to healthy control animals. Understanding the extent to which energy requirements and nutrient deposition change in pigs experiencing sustained immune stress may help develop more effective feeding strategies for health challenged herds and encourage appreciation for the economic benefits of maintaining high health populations.

## Additional files

Additional file 1: **Table S1.** Pre-test diet ingredient and analyzed nutrient composition. Table provides ingredient and nutrient composition of the common, pre-test diet all pigs were fed prior to initiating experiment. (DOCX 17 kb)

Additional file 2: **Table S2.** Effect of treatment on pre- and post-challenge complete blood count values. Table provides LS means, time by treatment *P*-values, time *P*-values, and treatment *P*-values, as well as means comparisons results for complete blood count response variables. (DOCX 20 kb)

Additional file 3: **Table S3.** Effect of treatment on serum glucose, insulin, acute phase protein, and cytokine concentrations. Table provides LS means, time by treatment *P*-values, time *P*-values, and treatment *P*-values, as well as means comparisons results for serum glucose, insulin, acute phase protein, and cytokine response variables. (DOCX 26 kb)

## Abbreviations

IL-1α: Interleukin-1-alpha
TNFα: Tumor necrosis factor-alpha
AA: Amino acid
ADF: Acid detergent fiber
ADFI: Average daily feed intake
ADG: Average daily gain
AHP: Activity heat production
APP: Acute phase protein
ATTD: Apparent total tract digestibility
BW: Body weight
CBC: Complete blood count
CON: Control treatment
CP: Crude protein
DE: Digestible energy
DM: Dry matter
DMI: Dry matter intake
EE: Acid hydrolyzed ether extract
ENZ: Enzyme treatment
FHP: Fasting heat production
FHP_total_: Total fasting heat production
FIIR: Feed-induced immune response
GE: Gross energy
GM-CSF: Granulocyte macrophage colony-stimulating factor
HI: Heat increment
HP: Heat production
HP_10_: Heat production over 10 h post-challenge
HP_low_: Average of 10 lowest HP values over 10 h post-challenge
HP_total_: Total heat production
IL: Interleukin
IL-1ra: Interleukin-1-receptor antagonist
ISS: Immune system stimulation treatment
k_mg_: Energy efficiency for maintenance and growth
LD: Lipid deposition
LPS: Lipopolysaccharide
MBL: Mannose binding lectin
ME: Metabolizable energy
ME_m_: Metabolizable energy used for maintenance
N: Nitrogen
NDF: Neutral detergent fiber
NE: Net energy
NR: Nitrogen retention
PD: Protein deposition
RE: Retained energy
RE_l_: Retained energy as lipid
RE_p_: Retained energy as protein
RFI: Residual feed intake
RQ: Respiratory quotient
RQ_fast_: Respiratory quotient during the fasting period of heat production measurements
RQ_fed_: Respiratory quotient during the fed period of heat production measurements
TEF: Thermic effect of feeding
VCO_2_: Volume of carbon dioxide produced
VO_2_: Volume of oxygen consumed
WBC: White blood cell
