# Supplementary material for: Lipopolysaccharide immune stimulation but not β-mannanase supplementation affects maintenance energy requirements in young weaned pigs
Source: J Anim Sci Biotechnol. 2018 Jun 15;9:47. doi: 10.1186/s40104-018-0264-y (PMC6003148; doi:10.1186/s40104-018-0264-y)
Supplement: Supplementary file 1 — Table S1. Pre-test diet ingredient and analyzed nutrient composition. Table provides ingredient and nutrient composition of the common, pre-test diet all pigs were fed prior to initiating experiment. (DOCX 17 kb) [file 40104_2018_264_MOESM1_ESM.docx]

**TABLE S1.** Pre-test diet ingredient and analyzed nutrient composition

| Item | % of diet (as-fed basis) |
| --- | --- |
| Ingredient |  |
| Corn | 79.89 |
| Soybean meal (dehulled, solvent extracted) | 7.00 |
| Casein | 5.00 |
| Fish meal | 5.00 |
| Soybean oil | 0.50 |
| Limestone | 1.05 |
| Monocalcium Phosphate | 0.20 |
| Lysine HCL | 0.37 |
| L-Threonine | 0.15 |
| L-Tryptophan | 0.06 |
| Vit. Premix^1^ | 0.33 |
| Mineral Premix^2^ | 0.20 |
| Salt | 0.25 |
| Analyzed composition |  |
| DM | 87.68 |
| GE, Mcal/kg | 4.01 |
| CP | 17.56 |
| EE^3^ | 3.97 |
| Starch | 48.87 |
| NDF | 7.14 |
| ADF | 2.55 |
| β-mannan^4^ | 0.16 |
| endo-1,4-β-mannanase activity,^5^ | *Below detectable limit^6^* |

^1^Provided per kilogram of complete diet: 6,614 IU of vitamin A; 827 IU of vitamin D; 26 IU of vitamin E; 2.6 mg of vitamin K; 29.8 mg of niacin; 16.5 mg of pantothenic acid; 5.0 mg of riboflavin; 0.023 mg of vitamin B_12_.

^2^Provided per kilogram of complete diet: Zn, 165 mg as ZnSO_4_; Fe, 165 mg as FeSO_4_; Mn, 39 mg as MnSO_4_; Cu, 17 mg as CuSO_4_; I, 0.3 mg as Ca(IO_3_)_2_; and Se, 0.3 mg as Na_2_SeO_3_.

^3^Acid hydrolyzed ether extract

^4^β-mannan concentration calculated using values reported in Shastak et al. [69]

^5^endo-1,4-β-mannanase activity. One U = the amount of enzyme which generates 0.72 micrograms of reducing sugars per minute from a mannose-containing substrate at pH 7.0 and temperature of 40°C.

^6^The lowest detectable limit was 15,000 U/kg
