# Supplementary material for: Lipopolysaccharide immune stimulation but not β-mannanase supplementation affects maintenance energy requirements in young weaned pigs
Source: J Anim Sci Biotechnol. 2018 Jun 15;9:47. doi: 10.1186/s40104-018-0264-y (PMC6003148; doi:10.1186/s40104-018-0264-y)
Supplement: Supplementary file 2 — Table S2. Effect of treatment on pre- and post-challenge complete blood count values. Table provides LS means, time by treatment P-values, time P-values, and treatment P-values, as well as means comparisons results for complete blood count response variables. (DOCX 20 kb) [file 40104_2018_264_MOESM2_ESM.docx]

**TABLE S2.** Effect of treatment on pre- and post-challenge complete blood count values^1^

| Item | Pre-injection^2^ | | | |  | Post-injection^2^ | | | |  | *P*-value | | |
| --- | --- | --- | --- | --- | --- | --- | --- | --- | --- | --- | --- | --- | --- |
| Treatment | CON^3^ | ENZ^4^ | ISS^5^ | SEM |  | CON^3^ | ENZ^4^ | ISS^5^ | SEM |  | Time x Treatment | Time | Treatment |
| Cell type count^6^ (cells x10^9^/L) |  |  |  |  |  |  |  |  |  |  |  |  |  |
| Total cells | 464.1 | 432.6 | 436.5 | 43.3 |  | 430.0 | 426.6 | 305.9 | 42.5 |  | 0.219 | 0.071 | 0.276 |
| WBC | 24.25^a^ | 20.60^ab^ | 22.37^ab^ | 1.35 |  | 24.22^a^ | 19.77^b^ | 7.01^c^ | 1.33 |  | <.0001 | <.0001 | <.0001 |
| Neut | 6.53^a^ | 6.75^a^ | 6.33^a^ | 0.76 |  | 7.27^a^ | 6.82^a^ | 2.57^b^ | 0.78 |  | 0.007 | 0.091 | 0.017 |
| Bands | 0.200 | 0.294 | 0.234 | 0.049 |  | 0.296 | 0.189 | 0.409 | 0.126 |  | 0.308 | 0.676 | 0.553 |
| Eos | 0.242 | 0.229 | 0.355 | 0.105 |  | 0.170 | 0.248 | 0.045 | 0.047 |  | 0.221 | 0.137 | 0.558 |
| Baso | 0.044 | 0.051 | 0.149 | 0.050 |  | 0.172 | 0.096 | 0.046 | 0.049 |  | 0.194 | 0.101 | 0.880 |
| Lymph | 15.17^a^ | 12.65^ab^ | 12.50^ab^ | 1.25 |  | 15.58^a^ | 11.29^b^ | 3.91^c^ | 1.23 |  | <.0001 | <.0001 | 0.001 |
| Mono | 0.741^ab^ | 0.685^ab^ | 0.738^ab^ | 0.159 |  | 0.593^b^ | 1.058^a^ | 0.034^c^ | 0.155 |  | 0.006 | 0.212 | 0.023 |
| RBC | 7.42 | 7.46 | 7.78 | 0.16 |  | 7.30 | 7.08 | 7.40 | 0.16 |  | 0.057 | <.0001 | 0.328 |

^a-c^Within a row, treatment means without a common superscript differ, *P* < 0.05.

^1^n = 10 pigs per treatment per time period (pre- and post- challenge)

^2^Effect of treatment before (pre- challenge, d 8) and after (post- challenge, d 10) receiving the first intramuscular injection of either saline or lipopolysaccharide on d 10 of the experiment. Serum was collected at 1400 h each day (4 h post- challenge on d 10).

^3^Control treatment (CON) = pigs fed basal diet (0.0% β-mannanase) with saline injection.

^4^Enzyme treatment (ENZ) = pigs fed enzyme diet (0.056% β-mannanase) with saline injection.

^5^Immune system stimulation treatment (ISS) = pigs fed enzyme diet (0.056% β-mannanase) with LPS (*Escherichia coli* serotype O55:B5) injection.

^6^Basophils (Baso); eosinophils (Eos); immature neutrophils (Bands); lymphocytes (Lymph); mature neutrophils (Neut); monocytes (Mono); white blood cells (WBC)
