# Supplementary material for: Lipopolysaccharide immune stimulation but not β-mannanase supplementation affects maintenance energy requirements in young weaned pigs
Source: J Anim Sci Biotechnol. 2018 Jun 15;9:47. doi: 10.1186/s40104-018-0264-y (PMC6003148; doi:10.1186/s40104-018-0264-y)
Supplement: Supplementary file 3 — Table S3. Effect of treatment on serum glucose, insulin, acute phase protein, and cytokine concentrations. Table provides LS means, time by treatment P-values, time P-values, and treatment P-values, as well as means comparisons results for serum glucose, insulin, acute phase protein, and cytokine response variables. (DOCX 26 kb) [file 40104_2018_264_MOESM3_ESM.docx]

Table S3. Effect of treatment on serum glucose, insulin, acute phase protein, and cytokine concentrations^1^

| Item | Pre- challenge^2^ | | | |  | Post- challenge^2^ | | | |  | *P*-value | | | |
| --- | --- | --- | --- | --- | --- | --- | --- | --- | --- | --- | --- | --- | --- | --- |
| Treatment | CON^3^ | ENZ^4^ | ISS^5^ | SEM |  | CON^3^ | ENZ^4^ | ISS^5^ | SEM |  | Time x Treatment | Time | Treatment | |
| Glucose, mmol/L | 7.61 | 7.45 | 7.37 | 0.37 |  | 6.83 | 7.23 | 6.87 | 0.24 |  | 0.628 | 0.042 | 0.797 | |
| Insulin, pmol/L | 89.30 | 83.80 | 82.90 | 9.01 |  | 71.77 | 117.10 | 106.89 | 16.83 |  | 0.197 | 0.265 | 0.320 | |
| Insulin:Glucose | 11.68 | 10.84 | 11.32 | 0.96 |  | 10.74 | 15.88 | 15.58 | 1.97 |  | 0.235 | 0.076 | 0.181 | |
| Acute phase protein, mg/mL |  |  |  |  |  |  |  |  |  |  |  |  |  | |
| Haptoglobin | 1.65 | 0.92 | 1.11 | 0.20 |  | 0.990 | 0.826 | 0.895 | 0.199 |  | 0.148 | 0.008 | 0.225 | |
| MBL^6^ | 126.2 | 116.0 | 119.1 | 7.9 |  | 139.0 | 133.7 | 151.5 | 16.6 |  | 0.705 | 0.049 | 0.729 | |
| Cytokine, pg/mL^7^ |  |  |  |  |  |  |  |  |  |  |  |  |  |  |
| GM-CSF | 15.88^ab^ | 17.44^ab^ | 11.67^b^ | 10.73 |  | 6.84^b^ | 4.84^b^ | 46.45^a^ | 10.59 |  | 0.001 | 0.412 | 0.333 | |
| IL-1α | 32.05 | 27.78 | 12.26 | 16.81 |  | 19.61 | 25.28 | 16.53 | 16.70 |  | 0.296 | 0.411 | 0.837 | |
| IL-1β | 1090.1^b^ | 913.8^b^ | 1361.6^b^ | 460.8 |  | 923.8^b^ | 375.2^b^ | 2588.8^a^ | 460.0 |  | 0.007 | 0.438 | 0.087 | |
| IL-1ra | 449.0^b^ | 853.0^b^ | 490.0^b^ | 365.7 |  | 448.2^b^ | 501.2^b^ | 108752.0^a^ | 7528.7 |  | <.0001 | <.0001 | <.0001 | |
| IL-2 | 328.2 | 324.7 | 133.0 | 137.6 |  | 320.3 | 123.2 | 92.0 | 135.9 |  | 0.449 | 0.220 | 0.505 | |
| IL-4 | 980.6 | 983.5 | 439.7 | 468.9 |  | 1089.6 | 416.1 | 241.3 | 463.1 |  | 0.492 | 0.342 | 0.531 | |
| IL-6 | 171.0^b^ | 123.1^b^ | 407.5^b^ | 216.1 |  | 136.8^b^ | 47.9^b^ | 1182.3^a^ | 214.7 |  | <.0001 | 0.001 | 0.051 | |
| IL-8 | 268.4^b^ | 400.1^b^ | 404.2^b^ | 83.8 |  | 240.0^b^ | 302.6^b^ | 1978.2^a^ | 330.3 |  | 0.001 | 0.009 | 0.006 | |
| IL-10 | 521.4 | 433.5 | 232.3 | 146.0 |  | 261.9 | 168.6 | 282.3 | 74.6 |  | 0.052 | 0.013 | 0.671 | |
| IL-12 | 1525 | 1789 | 1672 | 147 |  | 1646 | 1798 | 2093 | 145 |  | 0.073 | 0.020 | 0.302 | |
| IL-18 | 1953 | 1645 | 1114 | 455 |  | 1571 | 917 | 1213 | 448 |  | 0.431 | 0.217 | 0.546 | |
| TNFα | 23.7^b^ | 19.8^b^ | 74.9^b^ | 36.5 |  | 35.69^b^ | 12.47^b^ | 662.14^a^ | 110.82 |  | 0.005 | 0.016 | 0.001 | |

^a,b^Within a row, treatment means without a common superscript differ, *P* < 0.05.

^1^n = 10 pigs per treatment per time period (pre- and post-challenge)

^2^Effect of treatment before (pre-challenge, d 8) and after (post- challenge, d 10) receiving the first intramuscular injection of either saline or lipopolysaccharide on d 10 of the experiment. Serum was collected at 1400 h each day (4 h post- challenge on d 10).

^3^Control treatment (CON) = pigs fed basal diet (0.0% β-mannanase) with saline injection.

^4^Enzyme treatment (ENZ) = pigs fed enzyme diet (0.056% β-mannanase) with saline injection.

^5^Immune system stimulation treatment (ISS) = pigs fed enzyme diet (0.056% β-mannanase) with LPS (*Escherichia coli* serotype O55:B5) injection.

^6^Mannose binding lectin A (MBL)

^7^Granulocyte-macrophage colony-stimulating factor (GM-CSF); interleukin-1α (IL-1α); interleukin-1β (IL-1β); interleukin-1 receptor antagonist (IL-1ra); interleukin-2 (IL-2); interleukin-4 (IL-4); interleukin-6 (IL-6); interleukin-8 (IL-8); interleukin-10 (IL-10); interleukin-12 (IL-12); interleukin-18 (IL-18); tumor necrosis factor alpha (TNFα)
